# Colon-Targeted Delivery of Indole Acetic Acid Helps Regulate Gut Motility by Activating the AHR Signaling Pathway

**DOI:** 10.3390/nu15194282

**Published:** 2023-10-08

**Authors:** Ying Chen, Ruili Pan, Liya Mei, Peijun Tian, Linlin Wang, Jianxin Zhao, Wei Chen, Gang Wang

**Affiliations:** 1 State Key Laboratory of Food Science and Resources, Jiangnan University, Wuxi 214122, China; manju_ying@163.com (Y.C.); ruili_pan1222@163.com (R.P.); 6190112082@stu.jiangnan.edu.cn (L.M.); pjtian@jiangnan.edu.cn (P.T.); wanglinlin@jiangnan.edu.cn (L.W.); zhaojianxin@jiangnan.edu.cn (J.Z.); chenwei66@jiangnan.edu.cn (W.C.); 2 School of Food Science and Technology, Jiangnan University, Wuxi 214122, China; 3 National Engineering Research Center for Functional Food, Jiangnan University, Wuxi 214122, China; 4 (Yangzhou) Institute of Food Biotechnology, Jiangnan University, Yangzhou 225004, China

**Keywords:** chitosan, Eudragit S-100, drug delivery, indole acetic acid, gut motility, aryl hydrocarbon receptor

## Abstract

Intestinal peristalsis is vital for gastrointestinal physiology and host homeostasis and is frequently dysregulated in intestinal disorders. Gut microbiota can regulate gut motility, especially through the tryptophan metabolism pathway. However, the role of indoles as microbial tryptophan metabolites in colonic function requires further exploration. Here, we show that the delivery of indole acetic acid (IAA) targeting the colon can improve gut motility by activating the aryl hydrocarbon receptor (AHR). To achieve colon-targeted delivery, Eudragit S-100 (ES) and chitosan (CS) were used as drug carriers. After optimisation, IAA-loaded ES-coated CS nanoparticles exhibited an encapsulation efficiency of 83% and a drug-loading capacity of 16%. These nanoparticles exhibited pH-dependent characteristics and remained stable in acidic conditions and the upper intestine. In simulated intestinal fluid (pH 7.4) and colonic lumen, considerable amounts of IAA were released after approximately 4 h. Compared with free IAA, the nanoparticles exerted enhanced therapeutic effects on gut movement disorders induced by loperamide. The efficacy of IAA treatment was attributable to the activation of the AHR signalling pathway and increased levels of AHR agonists. Furthermore, the oral administration of IAA-loaded nanoparticles promoted serotonin secretion and maintained the intestinal barrier function. The experimental outcomes demonstrate the efficiency of the proposed colon-specific delivery system and highlight the role of IAA, produced by gut microbiota metabolism, in regulating gut peristalsis through AHR activation.

## 1. Introduction

Intestinal peristalsis is a crucial physiological process for gastrointestinal physiology and host defence mechanisms [1]. It is responsible for the movement of food and waste materials through the digestive system, ensuring the proper digestion and absorption of nutrients, as well as the elimination of waste products [2,3]. The dysregulation of intestinal peristalsis is frequently observed in various intestinal disorders, leading to symptoms such as constipation or diarrhoea [4,5]. The regulation of gut motility is a complex process that involves the interplay of neural, hormonal, and microbial influences. In recent years, the role of gut microbiota in modulating intestinal peristalsis has attracted considerable attention [6,7]. Evidence suggests that the depletion of intestinal microbiota may result in slow gut motility and an increased intestinal transit time (ITT) [8]; however, its underlying mechanism remains unclear. The regulation of intestinal peristalsis via the gut microbiota may depend on direct cell-to-cell interactions or the effect of metabolites. In this context, the influence of bacterial metabolites on intestinal physiology requires additional exploration.

Aryl hydrocarbon receptor (AHR) signalling in enteric neurons has been proven to be a regulatory node of intestinal peristalsis. The neuron deletion of AHR can reduce colonic motility, while AHR activation can partially restore intestinal movement and shorten ITT, as demonstrated in mice treated with antibiotics [9]. Several microbial indoles, such as indole acetic acid (IAA), indole acrylic acid (IA), indole-3-carboxaldehyde (ICA), tryptamine (TA), indole propionic acid (IPA), and indole lactic acid (ILA), have been identified as ligands for AHR [10,11]. Tryptophan can be transformed by intestinal microorganisms into indole and its derivatives. The imbalance of bacterial tryptophan catabolites has been observed in colonic motility disorders [12]. In addition, indole-3-carbinol, structurally similar to bacterial indoles, has been noted to decrease ITT and mediate gut peristalsis through the AHR signalling pathway, suggesting the potential role of indole derivatives in regulating intestinal peristalsis. Therefore, indoles within the gut can potentially enhance gut motility. Moreover, the distribution of AHR in intestinal cells depends on microbiota colonisation, and thus, AHR signalling is stronger in the colon than in the small intestine. Hence, targeting AHR in the colon may be a more effective approach for improving gut peristalsis.

As a known high-affinity xenobiotic ligand for AHR, IAA has a higher concentration than other indole derivatives [13,14]. Thus, it is expected to exert the most notable influence and can be used as a representative compound to investigate the role of intestinal indoles. Notably, IAA is a small molecule and fat-soluble substance which can be easily absorbed by the small intestine. In this context, a colon-specific delivery system may be beneficial for improving the bioavailability of IAA and simulating enterogenous metabolites in the colon. Chitosan (CS) is a potent carrier vehicle owing to its nontoxicity, biocompatibility, biodegradability, and low immunogenicity [15]. Ionic gelation is most frequently used for synthesising CS nanoparticles [16,17]. In particular, the nanoparticles formed by CS and tripolyphosphate (TPP) have been identified as a remarkable carrier for target therapy. However, CS is vulnerable to degradation in acidic conditions, which may result in excessive drug release in the stomach and upper intestine when administered orally [18]. Eudragit^®^ S-100 (ES), a pH-dependent synthetic polymer, has been widely applied in colon-targeted drug delivery systems [19,20]. ES can function as an enteric coating, thereby protecting CS nanoparticles from degradation in acidic environments [21]. Both materials rapidly degrade in the colon, and thus, a dual-coating approach involving both materials can mitigate drug release before entering the colon.

Therefore, this study aimed to establish an IAA-loaded colon-specific delivery system combining CS and ES coatings to increase the efficiency of oral IAA at the target site. The effects of IAA on intestinal peristalsis through the activation of the AHR signalling pathway were investigated. Targeting delivery could prevent IAA absorption through the small intestine—which can weaken AHR signalling—thereby improving IAA’s bioavailability.

## 2. Materials and Methods

### 2.1. Materials

CS (low viscosity, <200 mPa·s), 100 kDa CS (degree of deacetylation of 99%), and sodium TPP were purchased from Aladdin Biochemical Technology Co., Ltd. (Shanghai, China). High-molecular-weight CS (150 kDa) and β-glucosidase were obtained from Macklin Biochemical Technology Co., Ltd. (Shanghai, China). Eudragit^®^ S100 was purchased from Evonic Specialty Chemicals Co., Ltd. (Weiterstadt, Germany). Indole-3-acetic acid (analytical standard) was obtained from Sigma-Aldrich. Carmine red dye, methylcellulose, pepsin, trypsin, and various antibiotics were sourced from Sangon Biotech Technology Co., Ltd. (Shanghai, China). All antibodies were provided by ABclonal Technology Co., Ltd. (Wuhan, China). Loperamide hydrochloride (2 mg per capsule) was purchased from Xi’an Janssen Pharmaceutical Ltd. (Xi’an, China). All other reagents used in this study were of an analytical or high-performance liquid chromatography (HPLC) grade.

### 2.2. Preparation of Nanoparticles

The IAA-loaded nanoparticles were prepared using a modified version of the method developed by Pereira et al. [22]. Different CS solutions (3 mg/mL) were prepared by dissolving CS in 1% acetic acid (*w*/*v*) under stirring at 25 °C until the solution became transparent. The solution’s pH was adjusted to 5.3 using 10 mol/L NaOH. A dropwise addition of IAA solution (2 mg/mL in ethanol) was operated into the CS solution under magnetic stirring (400 rpm). After 1 h, a TPP aqueous solution (1 mg/mL) was added dropwise to the mixture with mild stirring (600 rpm) for another hour (400 μL/min). The formation of IAA-loaded nanoparticles was based on ionotropic gelation between CS and TPP. The CS/TPP weight ratio was 4:1, and the mass ratio of CS to IAA varied from 9:1 to 3:1. To prepare the ES-coated CS nanoparticles, a solution (1 mg/mL, ES dissolved in ethanol) was slowly added to the CS–TPP solution and stirred (850 rpm) overnight, with the ES/CS mass ratio at 9:1. Subsequently, the mixture was centrifuged at 35,000× *g* for 10 min, and the pellet was washed two times using ethanol to remove any free drugs. The obtained nanoformulations were lyophilised and stored at −20 °C.

### 2.3. Characterisation of the Nanoparticles

The particle size, zeta potential and polydispersity index (PDI) of the nanoparticles were measured using a Zetasizer nano ZS device (Malvern Instruments Ltd., Worcestershire, UK). The initial sample was diluted 10-fold with ultrapure water before testing, and all measurements were obtained in triplicate.

### 2.4. Encapsulation and Loading Efficiencies of the Nanoparticles

The free IAA in the supernatant was quantified using a Waters e2695 HPLC system with a 2475 Multi λ fluorescence detector (Waters, Milford, MA, USA). After undergoing centrifugation and two cycles of washing with methanol, the combined supernatant was placed at −20 °C for 30 min and then centrifuged at 12,000× *g* for 10 min. The extracted supernatant was passed through a 0.22 μm filter membrane, and the IAA levels in the effluent were determined using HPLC. Chromatographic separations were performed on a Waters Atlantis T3 (250 mm × 4.6 mm, 5 μm) column using a binary gradient (solvent A: 0.01 mol/L phosphate-buffered saline (PBS), pH 4.0; solvent B: acetonitrile). The following gradient was used: 5% B, 0–1 min; 95% B, 1–12 min; 95%, 12–15 min; 5% B, 15–16 min; and 5% B, 16–20 min. The flow rate was 1 mL/min, and the column temperature was set as 37 °C. The injection volume was 10 μL. The excitation and emission wavelengths for IAA detection were 280 nm and 360 nm, respectively. Subsequently, the encapsulation efficiency (EE) and drug loading (DL) were calculated using the following equations:

(1) $$EE\%=\frac{amout\;of\;total\;IAA-amout\;of\;free\;IAA\;in\;supernatant}{total\;amout\;of\;IAA}\times 100$$

(2) $$DL\%=\frac{amout\;of\;total\;IAA-amout\;of\;free\;IAA\;in\;supernatant}{weight\;of\;nanoparticles}\times 100$$

### 2.5. In Vitro Release of IAA from the Nanoparticles

The release of IAA from nanoparticles in various simulated digestive fluids was realised using previously reported methods [23]. Specifically, IAA-loaded nanoparticles were incubated in simulated gastric fluid (10 mg/L pepsin in 0.1 mol/L HCl, pH 1.2) for 4 h, simulated intestinal fluid (10 mg/L trypsin in PBS, pH 6.8) for 4 h and simulated colonic fluid (10 mg/L β-glucosidase in PBS, pH 7.4) for 12 h under magnetic stirring. At predetermined time intervals, 0.1 mL of the mixture was sampled, and the IAA content in the solution was quantified using HPLC, as described above. The corresponding IAA release percentage was calculated using the following equation:

(3) $$Release\%=\frac{amout\;of\;IAA\;released}{total\;amout\;of\;IAA\;loaded\;in\;the\;nanoparticles}\times 100$$

### 2.6. In Vivo Release and Biodistribution of IAA

**Figure 1:**
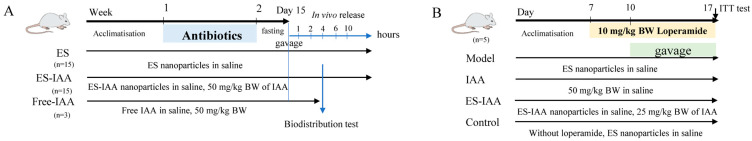
Study design timeline. (**A**) In vivo release and biodistribution test; (**B**) The treatment experiment.

Female Sprague–Dawley (SD) rats (7 weeks old, weighing 180–200 g) were purchased from Weitonglihua Experimental Animal Tech Co. (Beijing, China). All procedures involving animal subjects adhered to the Institutional Animal Care and Use Committee guidelines (Jiangsu, China) and were approved by the Ethics Committee of Experimental Animals at Jiangnan University (JN No. 20220615S0880902 [224]). The experimental schedule is described in Figure 1A. To deplete gut microbiota and the associated metabolite IAA, the rats were administered with an antibiotic solution consisting of 1 g/L of neomycin sulphate, ampicillin, and metronidazole; 0.5 g/L vancomycin hydrochloride, and 0.1 g/L acesulfame potassium in 4 mM acetic acid for a week. Normal drinking water was provided one day before the next experiment. Following this treatment, rats were randomly allocated into two groups (*n* = 15): the ES-IAA group and the ES group. Specifically, 1 mL of IAA-loaded ES nanoparticles (ES-IAA, IAA dose: 50 mg/kg body weight, BW) or IAA-free ES nanoparticles were administered orally to rats that had fasted overnight. At predetermined sampling intervals (*t* = 1 h, 2 h, 4 h, 6 h and 10 h), the contents of the stomach, small intestine and colon were collected (*n* = 3) from each group. The contents were lyophilised, weighed and then mixed with 900 μL MeOH/H_2_O (1:1). After homogenisation and centrifugation, the supernatant was concentrated to evaporate the solvent. The resulting samples were dissolved in 100 μL MeOH/H_2_O (1:9) and filtered using a 0.22 μm syringe filter for HPLC analysis. For the biodistribution study, rats were separately administered with ES-IAA nanoparticles and free IAA (*n* = 3, IAA dose: 50 mg/kg BW). After 4 h, the liver, kidney, spleen, lung, stomach, small intestine, cecum, colon, brain, hepatic portal vein, and arterial blood were collected, and the IAA concentrations were determined using HPLC. The pretreatment of tissues and organs was performed, as described earlier [13].

### 2.7. Treatment Study Design

After acclimatisation for one week, 20 rats were randomly divided into four groups as the model, control, IAA group and ES-IAA group (*n* = 5) and subjected to an intragastric administration of loperamide (10 mg/kg BW) for three days, excluding the control group. Subsequently, as shown in Figure 1B, the rats in the model and control groups received 1 mL of an ES nanoparticles saline solution via gavage for one week. The IAA group was administered 50 mg/kg BW of IAA in saline, and the ES-IAA group received an ES-IAA (IAA dose: 25 mg/kg BW) saline solution. During this one-week period, the model and intervention groups were administered loperamide one hour before IAA intake. Faecal samples were collected after one week of intervention. These samples were weighed before and after they were dried through heating, and the faecal water content was measured.

### 2.8. Assay of ITT

The ITT was determined as previously described [9]. At 09:00, all rats were orally administered 200 μL of 6% (*w*/*v*) carmine red dye in 0.5% (*w*/*v*) methylcellulose. The time from gavage until the appearance of the first red pellet was recorded as the ITT. The small intestinal transit rate was calculated as the percentage of the maximum distance reached by the carmine red dye relative to the total length of the small intestine. Before the experiment, the rats underwent fasting for 12 h, followed by the administration of 200 μL of a carmine red dye solution via gavage. After 30 min, the rats were euthanised, and the distance travelled by the dye was recorded.

### 2.9. Quantitative Polymerase Chain Reaction (PCR)

The total RNA from the colon was extracted using the TRIzol method and a HiScript III 1st Strand cDNA synthesis kit (Vazyme, Nanjing, China) to obtain complementary DNA. A quantitative PCR was implemented using SYBR Green Supermix and Bio-Rad CFX384 (Bio-Rad, Hercules, CA, USA). The cycle threshold (*C_t_*) values were normalised to β-actin. The primer sequences used in this study are summarised in Table 1.

### 2.10. Western Blot Analysis

Tissues were lysed in an RIPA lysis buffer containing 1 mM phenylmethylsulfonyl fluoride (PSMF). Following homogenisation and centrifugation, the total protein concentration in the supernatant was quantified using the BCA protein assay kit (Sangon). Subsequently, 30 μg of the protein extracts were separated on 10% SDS-PAGE gel, transferred onto a membrane, and probed for the rabbit polyclonal antibody AHR (dilution 1:2000), CYP1A1 (dilution 1:2000), and β-actin (dilution 1:1000). The relative expression of the protein was analysed using ImageJ 1.8.0 software.

### 2.11. Ultrahigh-Performance Liquid Chromatography–Mass Spectrometry (UPLC-MS) Analysis of Tryptophan Metabolites

The sample preparation and measurement of tryptophan metabolites from colonic contents and plasma were performed as previously described [13]. The pretreated samples were filtered through a 0.22 μm syringe filter and subjected to UPLC-MS analysis. The concentrations of indole derivatives and 5-HT neurotransmitter were quantified using external standardisation.

### 2.12. Statistical Analysis

The statistical comparisons between ES and ES-IAA group or IAA and ES-IAA group were performed using Student’s *t*-test with the GraphPad Prism 9.4 tool. For multiple comparisons, a one-way analysis of variance was carried out with Dunnett’s multiple comparisons test. All values are presented as the mean ± standard deviation (SD) of at least three independent experiments. Statistical significance is indicated by an adjusted *p*-value < 0.05.

## 3. Results and Discussion

### 3.1. Characterisation of Nanoparticles

To construct IAA-loaded nanoparticles, a different CS was used to identify the CS type with the highest EE for IAA. As indicated in Table 2, no notable differences in particle size or zeta potential were observed among the three types of IAA-loaded CS nanoparticles. However, the EE and DL of low-viscosity CS-based nanoparticles were lower than those of the other CS nanoparticles, suggesting that low-viscosity CS cannot enhance drug loading. In comparison, 150 kDa CS-coated nanoparticles exhibited superior IAA encapsulation and DL capability. With increases in the amount of IAA added to the CS solution, the percentage of DL reached its maximum value of 18.18 ± 0.70% at the CS/IAA mass ratio of 4.5:1, with no increase in IAA contents observed at higher amounts. Thus, a 150 kDa CS and a CS/IAA ratio of 4.5:1 were selected to construct the colonic delivery system.

To ensure the efficiency of the colon-targeted delivery system, the pH-dependent soluble polymer ES was used to coat the CS nanoparticles to prevent their degradation in the stomach and upper intestine. Owing to the increase in the number of layers, ES-coated nanoparticles exhibited larger sizes than the non-coated nanoparticles (Table 2). Moreover, the presence of differently-sized particles led to an increase in PDI. Owing to the negative charge of ES, the zeta potential was lower than that of the corresponding CS nanoparticles. Additionally, the EE of ES-IAA nanoparticles was higher than that of the non-coated nanoparticles, indicating that some free IAA in the solution or on the surface of CS-IAA nanoparticles may have been coated using ES. Despite the added weight of ES nanoparticles, the average DL percentage in these nanoparticles showed little reduction.

### 3.2. In Vitro Release of IAA-Loaded Nanoparticles

**Figure 2:**
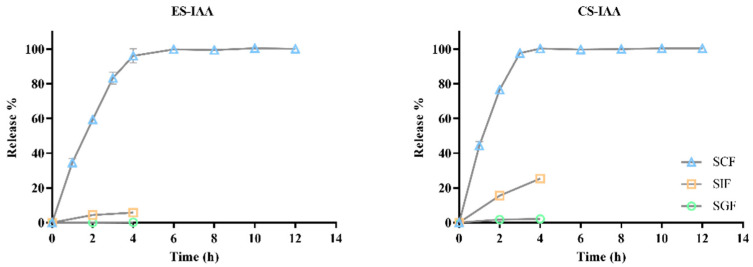
In vitro release profile of IAA from the ES-IAA and CS-IAA nanoparticles in different simulated mediums.

Food remains in the stomach, small intestine, and colon for approximately 2, 4, and 10 h, respectively. To investigate the release of CS-IAA and ES-IAA nanoparticles in gastroenteric environments, a simulated gastrointestinal fluid was used. The release profile of IAA in the simulated gastrointestinal tract is shown in Figure 2. In the first 4 h, only a small percentage of IAA from the ES-coated nanoparticles (<1%) was released into the simulated gastric fluid (SGF), whereas approximately 2% of IAA was detected in the CS-IAA mixture. Exposure to simulated intestinal fluid (SIF) for 4 h resulted in the release of approximately 6% of IAA from the ES-IAA nanoparticles. Additionally, the multi-layer structure consisting of ES and CS/TPP decreased the release rate of IAA from nanoparticles. In the absence of the ES coating, the release rate of IAA increased by approximately 25% in SIF. Following incubation with the simulated colonic fluid (SCF), CS-IAA nanoparticles rapidly released IAA. At pH 7.4, the deprotonation of CS weakened the interaction between CS and TPP, leading to the disintegration of CS nanoparticles. With the catalysis of β-glucosidase, the degradation of CS was accelerated. Thus, the percentage of IAA released from CS-IAA nanoparticles reached 100% at 4 h, with only 96% IAA quantified in the ES-IAA solution. Within 6 h, all the IAA was released from the ES-coated nanoparticles. Considering the higher IAA release from CS nanoparticles in the SGF and SIF, the combination of the ES and CS coating was selected to enhance IAA delivery to the colon.

### 3.3. In Vivo Release and Biodistribution of IAA

**Figure 3:**
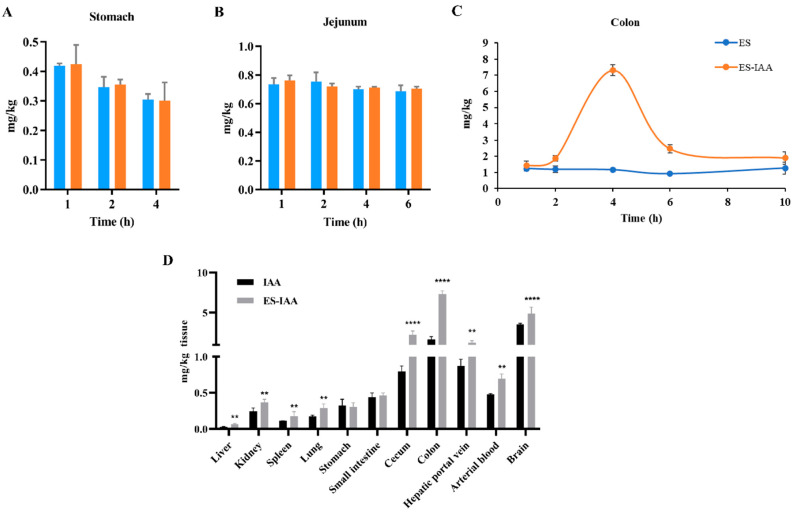
In vivo release and biodistribution of IAA. The detection of IAA in the stomach (**A**), jejunum (**B**) and colon (**C**) in rats treated with empty or IAA-loaded nanoparticles. The blue column is group ES, the orange column is group ES-IAA. (**D**) IAA contents in different tissues 4 h after oral gavage with free IAA and ES-IAA nanoparticles. Significance analysed by Student’s *t*-test (** *p* < 0.01 and **** *p* < 0.0001).

Following the optimisation of the coating matrix, the IAA-loaded delivery system targeting the colon was established. To evaluate the specificity and release characteristics, rats were administered ES-IAA nanoparticles. The rats were given an antibiotic treatment to remove most of the gut microbiota and reduce the accumulation of primary IAA. The measurement of IAA in the stomach contents showed no significant difference between rats administered empty or IAA-loaded ES nanoparticles (Figure 3A). This finding indicates that the ES film of the delivery system could ensure the structural integrity of nanoparticles in the harsh and acidic environment of the stomach. Upon progression through the upper intestine, the ES-IAA nanoparticles remained stable over time, and the IAA concentration in the jejunum did not exhibit a significant increase in the ES-IAA group compared with the control (Figure 3B). Moreover, the IAA level in the ileal contents was below the detection limit and could not be analysed using HPLC. The multi-layer structure prevented the early release of drugs into the small intestine and enhanced the efficiency of the colon-targeted delivery system. Two hours after gavage with ES-IAA nanoparticles, the IAA level in the colon began to increase, indicating the release behaviour of the nanoparticles. At 4 h, the release amount reached its peak (Figure 3C). At over 10 h, the IAA concentration was consistently higher than that in the control, suggesting a sustained release of drug-loaded nanoparticles.

An in vivo biodistribution trial was performed at 4 h after oral gavage when the high-level accumulation of IAA in the colon was observed. At this point, rats administered free IAA exhibited a higher concentration of IAA in the colon than antibiotic-treated rats (Figure 3D). Combined with the observation of low IAA levels in the contents of the stomach and small intestine, this outcome indicated that unabsorbed IAA entered the colon and increased drug accumulation (1.68 ± 0.28 mg/kg). However, the IAA concentration was significantly lower than that in rats dosed with IAA-loaded nanoparticles (7.32 ± 0.34 mg/kg), suggesting that ES-IAA nanoparticles were selectively degraded in the colon, thereby greatly increasing the IAA concentration at the target site. Moreover, the increased IAA content in plasma from the hepatic portal vein, resulting from colonic reabsorption, indicated the colon-specific release of these nanoparticles. Additionally, IAA, as a liposoluble substance, can cross the blood–brain barrier and enter the brain, where it may participate in neuromodulation. Therefore, further analysis of the gut–brain axis is warranted. Overall, the in vivo results demonstrate the high efficiency of the proposed IAA-loaded colonic delivery system and its potential in target therapy.

### 3.4. Effects of IAA Colonic Delivery on Gut Motility

**Figure 4:**
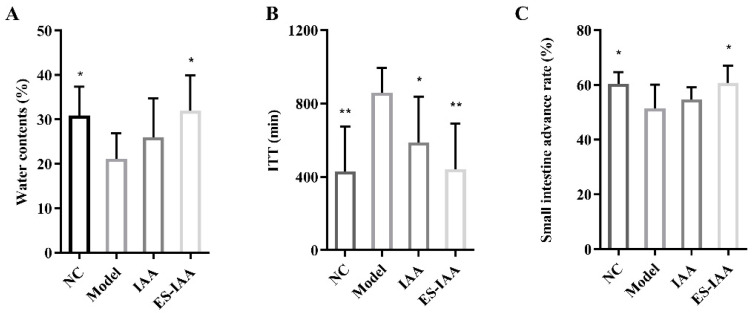
Effects of colon-targeting IAA on intestinal peristalsis. (**A**) Detection of water contents of faeces. (**B**) Quantification of the effect of IAA on total ITT. (**C**) Measurement of small intestinal transit rate in rats. one-way ANOVA with Dunnett’s multiple comparisons test was performed. * *p* < 0.05 and ** *p* < 0.01 compared with the model group.

To enhance the efficiency of IAA at the target site, an oral colon-specific delivery system was established. An animal model with gut motility disorders induced via loperamide was used to evaluate the effect of IAA on intestinal movement. Compared with rats in the control group, rats treated with loperamide presented lower water contents, prolonged colonic ITT, and a reduced small intestinal advance rate (Figure 4). Notably, the ES-IAA group showed significant improvements in these symptoms, while rats receiving empty nanoparticles did not display any amelioration of the gut motility disorder. This finding indicates that the nanocarrier itself is not involved in regulating gut peristalsis. Also, the oral administration of IAA (50 mg/kg) significantly reduced ITT. By contrast, loperamide-fed rats treated with IAA-loaded nanoparticles (25 mg/kg) could more efficiently alleviate the gut motility disorder. Overall, IAA could regulate intestinal motility and was more effective when delivered through a colon-targeted delivery system.

### 3.5. Regulation of Gut Movement through AHR Activation

**Figure 5:**
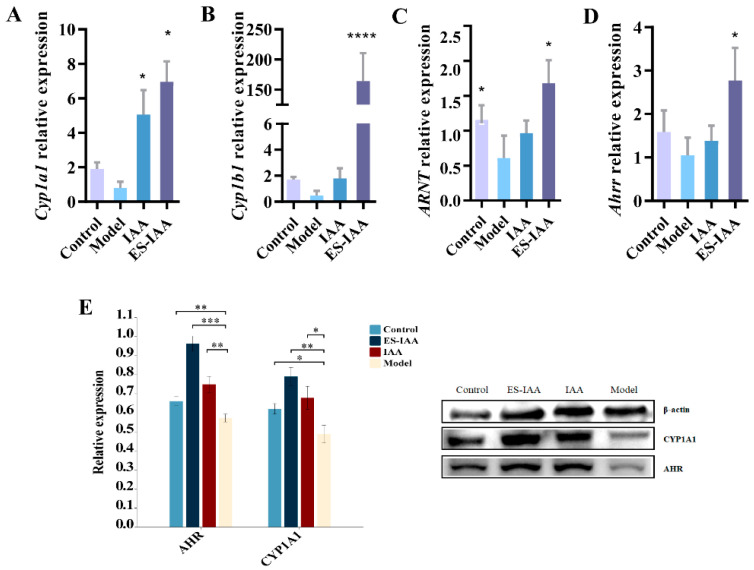
Activation of AHR signalling pathway in ES–IAA diet. The transcriptional level of *Cyp1a1* (**A**), *Cyp1b1* (**B**), *ARNT* (**C**) and *Ahrr* (**D**). Statistical analysis was carried out using one-way ANOVA with Dunnett’s multiple comparisons test, * *p* < 0.05, ** *p* < 0.01, *** *p* < 0.01 and **** *p* < 0.0001 compared with the model group. (**E**) The expression of protein CYP1A1 and AHR was detected by WB in different groups and relative expression was analysed using Image J.

To evaluate the effect of IAA as an AHR agonist on gut movement, the activation of the AHR signalling pathway was investigated. Increased transcriptional levels of the AHR nuclear transporter ARNT and target genes (*Cyp1a1*, *Cyp1b1*, and *Ahrr*) with elevated protein levels of CYP1A1 and AHR confirmed the activation of AHR signalling (Figure 5). However, the efficiency of IAA in improving gut movement differed between the IAA and ES-IAA groups. Correspondingly, the levels of AHR-related genes in the IAA group were slightly lower than those in the ES-IAA group, which could be correlated with the amount of IAA in the colon lumen.

**Figure 6:**
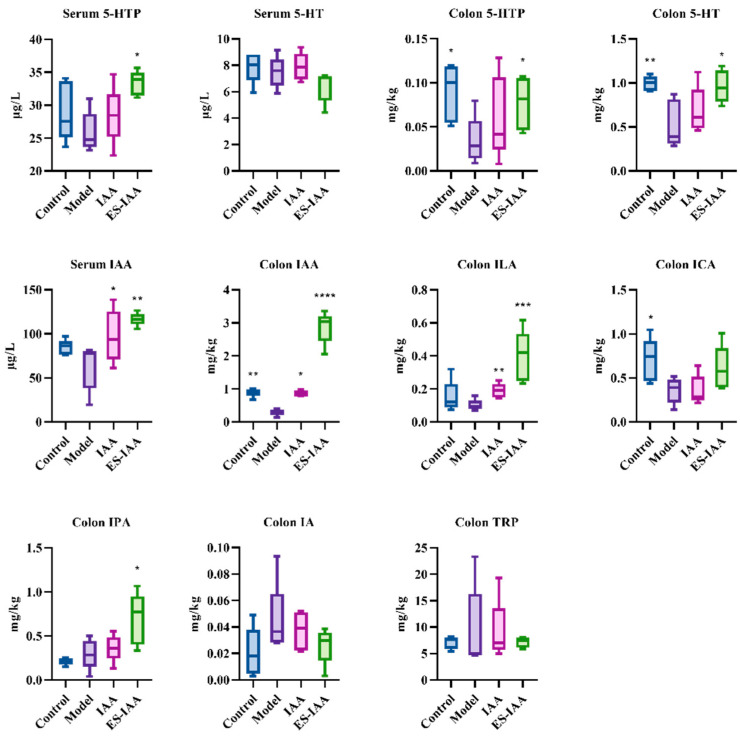
The concentration of 5-HT and microbial indoles in serum and colonic contents. Statistical analysis was performed using one-way ANOVA with Dunnett’s multiple comparisons test, * *p* < 0.05, ** *p* < 0.01, *** *p* < 0.001 and **** *p* < 0.0001 compared with the model group.

Furthermore, the accumulation of IAA in the colonic contents of rats administered ES-IAA nanoparticles was higher than that in the IAA group (Figure 6). Under IAA intervention, an increase in ILA and IPA formed via microbiota metabolism was observed. Simultaneously, the IAA and ICA levels decreased in rats with slow gut peristalsis induced by loperamide, indicating disruptions in tryptophan metabolism due to changes in the gut microbiota. Correspondingly, reductions in the CYP1A1 and AHR protein expression levels were present in the model compared with the control, which could aggravate slow colon transit. In addition, the levels of 5-hydroxytryptophan (5-HTP) and 5-hydroxytryptamine (5-HT) in the colon, which are secreted by enterochromaffin cells and can regulate gut peristalsis, significantly increased in the ES-IAA group. Overall, our experiments demonstrated how IAA could activate the AHR signalling pathway to regulate gut motility by providing additional AHR ligands.

### 3.6. mRNA Expression Levels of Mucin and Tight Junction (TJ)-Related Proteins

**Figure 7:**
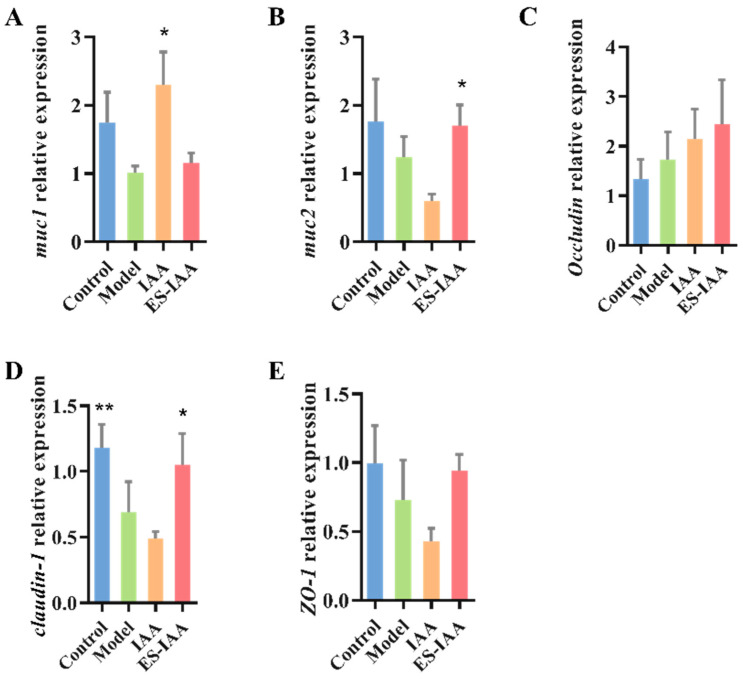
The relative expression of mucin and tight junction proteins affected by IAA. The mRNA levels of Muc1 (**A**), Muc2 (**B**), Occludin (**C**), Claudin-1 (**D**) and ZO-1 (**E**). Statistical analysis was carried out using one-way ANOVA with Dunnett’s multiple comparisons test compared with the model group, * *p* < 0.05 and ** *p* < 0.01.

The activation of the AHR signalling pathway is necessary for IAA to traverse the mucus layer and intestinal epithelial cells. To examine whether IAA treatment can influence the intestinal barrier functions, the mRNA levels of mucin 1 (*muc 1*), mucin 2 (*muc 2*), *occludin*, *claudin-1*, and zonula occludens-1 (*ZO-1*) in the colon were detected using a real-time PCR. The loperamide-induced model exerted a prominent decrease in the TJ protein *claudin-1* (Figure 7). No further disruption of the intestinal barrier was observed in either intervention group. However, the regulation of mucin and TJ protein mRNA levels in the IAA group was different from that in the ES-IAA group, suggesting a potential relationship with the location of the drugs in the colon or with the distance to target cells. Rats administered IAA-free nanoparticles exhibited enhanced mRNA levels of the transmembrane protein *muc 1*, while the ES-IAA group exhibited increased transcriptional levels of the secreted protein *muc 2*. Furthermore, the released IAA dramatically increased the *claudin-1* levels against loperamide treatment. These results suggest that an IAA-rich diet can protect colonic barrier function from loperamide-induced impairment through the activation of the AHR signalling pathway.

## 4. Discussion

An IAA-loaded colon-targeted delivery system combining CS and ES coatings was constructed in this study, which helped regulate gut motility more efficiently by more effectively activating the AHR signalling in the colon compared to free IAA intake. Colon-targeting released IAA and could stimulate the secretion of indole derivatives and 5-HT in the colon, maintaining colonic barrier function. This study provides a new proposal for treating the dysfunction of gut peristalsis more efficiently.

AHR is widely distributed in intestinal epithelial cells and enteric neuronal cells, with its amount depending on the abundance of microbiota. AHR signalling in enteric neurons has been identified as a regulatory mechanism for improving colonic peristaltic activity [9]. Several studies have reported that bacterial tryptophan metabolites, including IAA, IPA, IA, ILA, ICA, and TA, are ligands for AHR [10]. Moreover, the dietary intake of the AHR pro-ligand indole-3-carbinol can increase intestinal motility through the activation of AHR signalling [9]. IAA is a tryptophan metabolite produced by gut microbiota, which can be further metabolised into other indole derivatives [24]. It can exert effects on improving gut permeability, host immunity, insulin resistance, lipid metabolism and oxidative and inflammatory stress [25]. It was also reported that IAA could alleviate non-alcoholic fatty liver disease and modulate learning and memory in previous studies [26,27]. In addition, IAA was believed to suppress pro-inflammatory cytokine levels in macrophages and hepatocytes in an AHR-dependent manner [28]. However, the teratogenic potential of IAA at dose levels over 500 mg/kg can occur in mice and rats [29]. Also, IAA can be used as a drug to stop bleeding, pain and inflammation sterilisation, and general side effects, including dry skin and rashes [30].

Considering the role of AHR signalling in regulating gut motility and the fact that IAA can activate AHR signalling in mammals [9], IAA has great potential to regulate intestinal motility. However, this potential has not yet been proven. Hence, in this study, the effects of IAA treatment on the slow gut peristalsis induced by loperamide were explored. It should be noted that IAA, as a fat-soluble small molecule substance, is easily absorbed by the small intestine. To simulate the impact of indole metabolites produced by gut microbiota on colonic motility impairment, a colon-targeted delivery system could help IAA exert its effects.

CS polysaccharides have been widely used as a carrier for intestinal therapeutics owing to their biocompatibility, biodegradability, nontoxicity, and sustainable production [31]. In this study, CS and TPP were used to form the first layer via ionotropic gelation. Notably, the high viscosity of CS solutions can hinder the encapsulation of drugs by limiting the movement of drug molecules around the CS chain [32]. However, low-viscosity CS showed no superiority in the EE and DL compared to other CS materials in this study. Moreover, the CS/TPP coating may partially disintegrate when exposed to the acidic environment and the presence of pepsin, resulting in the early release of loaded drugs before entering the colon [21]. The pH-dependent synthetic polymer, Eudragit^®^ S-100 (ES), could also precisely prevent CS nanoparticles from experiencing degradation at a pH less than 7.0 [19], and this synthetic polymer has been widely applied in colonic targeting therapy for inflammatory bowel disease [20]. The interaction between CS and TPP can maintain the stability of the nanoparticles when the ES layer is exposed to a medium with a pH close to 7.0 and partially disintegrates. At pH 7.4, the deprotonation of CS weakens the interaction between CS and TPP, leading to the disintegration of CS nanoparticles. Indeed, in this study, the combination of CS and ES reduced IAA release in SGF and maintained the stability of IAA-loaded nanoparticles in the small intestine. On the other hand, in the colon, the intestinal epithelial cells secrete HCO3- into the mucin layer, thereby increasing the pH [26], and the microbiota of the mucus layer exhibit higher diversity than that in the lumen [29]. Hence, the pH-sensitive properties of the ES coating and microbial degradation of CS likely promote the release of IAA in the mucin layer. The release at this location could potentially facilitate the contact and entry of IAA into intestinal cells.

The colon-targeting delivered IAA in this study exhibited better effects on gut motility modulation and colonic barrier function protection than gavaged-free IAA. The function of IAA is achieved through the activation of AHR signalling. This study provides more evidence to support ligand-dependent AHR in protecting the intestinal barrier’s integrity and enhancing TJ proteins [33,34]. The regulation of mucin and TJ proteins via colon-targeting delivered IAA was different from that of gavaged-free IAA, suggesting the location of the medicine in the gastrointestinal tract or its distance from the target cells may result in various therapeutic effects. Another finding in this study is that IAA intervention can promote 5-HT secretion. It is known that the neurotransmitter 5-HT secreted by enterochromaffin cells can regulate gut peristalsis [35]. However, the relationship between 5-HT and IAA remains unclear, especially in terms of microbiota-dependent mechanisms or the AHR signalling pathway.

The IAA-loaded delivery system constructed in this study provides a colon-specific method for delivering small molecular substances and contributes to further investigations on the role of gut-derived molecules. In this study, IAA was also found in the brain, and its role in gut-brain axis remains unclear, which could achieve further understanding in future work.

## 5. Conclusions

We developed an IAA-loaded colon-targeted delivery system involving CS and ES coatings to eliminate the influence of small intestinal absorption and explore the role of IAA in the colon. IAA treatment significantly improved intestinal peristalsis and alleviated intestinal barrier dysfunction by activating the AHR signalling pathway. The proposed colon-targeted delivery system improved the therapeutic efficiency of IAA for gut motility disorders. Our research findings provide valuable insights into the role of IAA secreted by microbiota in the colon.

## Figures and Tables

**Table 1:** Primer sequences for real-time PCR.

Gene	Forward Primer (5′-3′)	Reverse Primer (5′-3′)
*Cyp1a1*	GCTGATGGCAGAGGTTG	ACGGAGGATAGGAATGAAG
*Cyp1b1*	CCGAAAAGAAGGCGACTGG	TGCACATCCGGGTATCTGGTAAAG
*ARNT*	AGAGACTTGCCAGGGAAAATCATA	TTTCGAGCCAGGGCACTACAGG
*Ahrr*	AAAGTCAGCATCCCTCCTTG	CCCATCAGATCCTTTGGATG
*Occludin*	AATGTAGAGAAAGGTCCTGGTG	CCTTTAATTCCTGCACCA
*Muc1*	TACCTACCACACTCACGGAC	TCCTACAAGTTGGCCGAAG
*Muc2*	CGCCAATTACGCTGAACACT	CCTCGTTGTTCTGACAGTTGC
*Claudin-1*	ACGCAGGAGCCTCGCCCCGCAGCTGCA	CAGCCAAGGCCTGCATAGCCATGG
*ZO-1*	CACACGATGCTCAGAGACGAAGG	CTGTATGGTGGCTGCTCAAGGTC
*Actb*	AGCCATGTACGTAGCCATCC	CTCTCAGCTGTGGTGGTGAA

**Table 2:** The characterisation of the nanoparticles in this study.

CS	Ratio	ES ^1^	d (nm)	PDI	Zeta (mV)	EE%	DL%
<200 mPa·s	9:1	-	196.10 ± 13.48	0.46 ± 0.03	+40.80 ± 1.39	71.06 ± 4.57	9.58 ± 0.62
100 kDa	9:1	-	186.70 ± 14.96	0.44 ± 0.05	+41.37 ± 1.06	74.67 ± 2.95	10.07 ± 0.40
150 kDa	9:1	-	201.23 ± 24.51	0.43 ± 0.03	+42.57 ± 3.90	80.17 ± 4.94	10.81 ± 0.62
150 kDa	4.5:1	-	185.83 ± 21.53	0.48 ± 0.02	+40.80 ± 1.67	71.94 ± 2.79	18.18 ± 0.70
150 kDa	4.5:1	+	297.33 ± 12.70	0.54 ± 0.10	+37.53 ± 0.66	83.15 ± 0.83	16.03 ± 0.16
150 kDa	3:1	+	301.43 ± 11.72	0.55 ± 0.01	+38.53 ± 1.44	66.3 ± 6.25	16.15 ± 1.52
150 kDa	-	+	251.83 ± 8.21	0.50 ± 0.05	+33.63 ± 0.94	-	-

^1^ ‘-’ was symbolled as no addition of materials or no detection, ‘+’ as addition of Eudragit^®^ S100.

## Data Availability

Not applicable.

## Abbreviation

IAA: indole acetic acid; AHR, aryl hydrocarbon receptor; ES, Eudragit S-100; CS, chitosan; ITT, intestinal transit time; TPP, tripolyphosphate; PDI, polydispersity index; EE, encapsulation efficiency; DL, drug loading; CS-IAA, IAA-loaded chitosan nanoparticle; ES-IAA nanoparticle, IAA-loaded Eudragit S-100-coated chitosan nanoparticle; ES nanoparticle, Eudragit S-100-coated chitosan nanoparticle; SGF, simulated gastric fluid; SIF, simulated intestinal fluid; SCF, simulated colonic fluid; 5-HTP, 5-hydroxytryptophan; 5-HT, 5-hydroxytryptamine.
